# PP40 Enoxaparin: Synthesis of Evidence for Expanding the Current National Clinical Protocol and Therapeutic Guidelines

**DOI:** 10.1017/S0266462325102596

**Published:** 2025-12-29

**Authors:** Gleidson Cardoso Spinola, Bárbara de Castro dos Santos Silva, Carolina Santos Silva, Manuela Fernandes de Almeida Mello, Rouseli Gonçalves de Menezes

## Abstract

**Introduction:**

Venous thromboembolism (VTE) prevention for women with additional risk factors during pregnancy is centered on anticoagulant therapy, with enoxaparin as the anticoagulant of fchoice. This study evaluated the efficacy, safety, and costs of enoxaparin for thromboprophylaxis in pregnant or postpartum women at risk for VTE, aiming to expand access to the Brazilian national Clinical Protocols and Therapeutic Guidelines (PCDT).

**Methods:**

A search was performed in the MEDLINE, LILACS, and Embase databases (January 2024) using a strategy that combined target population, enoxaparin, and low molecular weight heparin; filters for systematic review (SR) with or without meta-analysis; and publications up to five years, without language restriction. Duplicate exclusion and study eligibility were performed using Rayyan. Only eight studies met the pre-established criteria. For budget impact, enoxaparin values and doses, local hospital consumption, and epidemiological data were used. The daily, average, and annual costs of hospitalization of pregnant women (n=259) with VTE in the State of Bahia were calculated.

**Results:**

Results showed a reduction of thromboembolic events; a reduction of VTE in pregnant women at high perinatal risk as well as postpartum women who did not use anticoagulation in the antepartum period; an increased risk of hemorrhagic events in pregnant women, worsening during in vitro fertilization; a reduction in the risk of miscarriage and an increase in the rate of live births; and a significant increase in allergic reactions. Only one SR converged with the national guideline. The annual avoidable hospitalization cost was USD239,725.80, while the budget impact was USD163,207.47 in the first year. A group of experts was established to develop the state protocol with the expanded use of enoxaparin.

**Conclusions:**

The economic analysis showed a high average cost of prolonged hospitalization in pregnant women receiving enoxaparin. Thus, considering the High-Risk Pregnancy Manual after the PCDT, vulnerability, prevention of maternal morbidity and mortality, turnover of obstetric beds, reduction of hospitalization costs, and qualification of care, it was decided to develop a state protocol with expansion of anticoagulation indications.

